# Comparison of performance on written and OSCE assessment during end semester pediatric examination

**DOI:** 10.12669/pjms.36.4.2026

**Published:** 2020

**Authors:** Shazia Memon, Siraj-ul-Haque Shaikh

**Affiliations:** 1 Prof. Dr. Shazia Memon (FCPS, Pediatrics, MCPS–HPE). Professor of Pediatrics, Liaquat University of Medical and Health Sciences (LUMHS), Hyderabad, Sindh, Pakistan; 2 Dr. Sirajul Haque Shaikh, Director & Associate Professor, Department of Medical Education, College of Physicians & Surgeons Pakistan, Karachi, Pakistan

**Keywords:** Competencies, Cognitive domain, Psychomotor skills, Pediatrics

## Abstract

**Objective:**

To compare the scores obtained on written and OSCE examination during pediatrics end semester examination and to find out the reasons for the discrepancies among the scores obtained.

**Methods:**

This co-relational study was carried out in pediatric department, Liaquat University of Medical and Health Sciences (LUMHS). The performance of medical students who were posted in the pediatric department for one semester (November 2016 to October 2017) was compared on the basis of scores obtained in their written and OSCE during end semester examination. To find out the reasons for discrepancies qualitative data was collected by using likert scale on Pre-designed questionnaire distributed among the students at the end of assessment. Frequencies of their responses were calculated.

**Results:**

Data of 160 students who participated in study was analyzed by SPSS version 22. The mean and standard deviation of participant’s score was 51.25 ± 12.19. Females performed better in written as well as in OSCE. Moderate correlation was seen between MCQ and SAQ scores (r=0.5, p <0.01). Around 60% considered OSCE as their preferred mode of assessment. Deep learning approach and group discussion was found in female students (65%).

**Conclusion:**

Our study concluded that students performed better in OSCE than in written assessment. However, in OSCE they had shown unsatisfactory performance for some important competencies like clinical examination methods and procedural skills. Female students performed better in both segments of assessment.

## INTRODUCTION

Outcome based system of medical education, which is becoming progressively more common, requires policy makers to articulate standards based on outcomes for learner’s attainment, clearly defining the knowledge, skills, and attitudes learners are expected to achieve at different stages of their learning and upon graduation.^1^ Acquisition of an outcome implies that the graduate has the ability to apply the theoretical knowledge according to patient’s condition, and performing the clinical and practical skills accordingly. The training program specifically defines learning objectives to achieve those outcomes, and learner’s assessments should focus on attainment of those outcomes. Assessment is a process through which teachers analyze whether the educational outcomes of any particular course are achieved or not. If assessment tools do not appropriately match with the desired learning outcomes, then scores obtained on assessment have little value in judging the learner’s performance.^2^

For each learning outcome or competency to be performed the candidate needs some background knowledge, an appropriate skill and desirable behavior to perform the required task.^3^ Successful completion of undergraduate medical education, therefore, should provide its graduates with knowledge, skills and attitudes that help them achieve the desired outcomes for improving the health of community which they expected to serve.^4^ In undergraduate assessment the learning outcomes related to cognitive component are assessed during written assessment, case based discussions, drills and also bedside teaching including clinical reasoning and decision making skills; while the clinical skills are assessed in OSCE^5^, bed side teaching, and Mini-CEX. Harden first described the objective structured clinical examination (OSCE) in 1975 with the aim to evaluate clinical competencies of medical students in a comprehensive and consistent manner with reliability and objectivity of the process.^6^ Each student is required to demonstrate specific behaviors in a simulated work environment with strict control over the clinical context so as to keep it as close to the real tasks. On the other hand development and administration are time consuming, require standardization of simulated patients and examiners and also large number of assessors are required. 5

It is essential for medical institutions to train and evaluate its graduates according to the expected learning outcomes laid down in the curriculum.^1^ It is not surprising to find that many students performing well in cognitive assessment are weak in clinical skills.^7^ The group with inadequate skill acquisition proceeding further in the programme without an opportunity to further practice those skills, are likely to remain deficient. This state of affairs needs correction by introducing a system of remediation providing opportunity to practice clinical skill(s) even after completion of posting in the specialty. Introducing such an innovation is not an easy task and would require concrete data regarding average number and percentage of students failing in the skills component along with the reasons students attribute to their failure is needed to build a strong justification.^8^

The purpose of the study was to compare student’s performance in cognitive and psychomotor domains (including affects) upon completion of clinical posting in Pediatrics and to determine the reasons for the discrepancies, if found, so that some remedial mechanism could be recommended for filling those gaps particularly for adequate acquisition of basic clinical skills, which are essential for medical graduates in their future professional practice.

## METHODS

This co-relational study was conducted at Pediatric Department, Liaquat University of Medical and Health Sciences (LUMHS) Jamshoro, Sindh, Pakistan from November 2016 to October 2017. The scores of written tests and OSCE obtained by undergraduate students (batch 2012, 9^th^ & 10^th^ semester) during end semester pediatric examination were compared among each other. To find out the reasons for discrepancies students were given questionnaire at the end of assessment. Questionnaire was designed to assess the student’s perception regarding both assessment tools.

### Sample size and technique

Minimum sample required for this study was 132, (with perception of 5% as probable value of difference in the domain based achievement), 95% Confidence Interval, and 5% margin of error (WHO statistical manual), but 160 were included (80 from each semester) who were posted in the pediatric ward during the second half of their semesters. All the students who appeared in the formative tests and attended minimum of 75% lecture and ward posting classes were included, while the students whose attendance was less than 75%, or remained absent during formative assessments were excluded. The study was conducted after getting approval from research ethics committee LUMHS, Ref No. LUMHS/REC/471, dated January 20, 2016. All students who participated had given their consent to be the part of this study and their identity was kept confidential.

Dependent variable of our study were scores of students obtained in written test and OSCE, while Independent variables were gender, preferred mode of assessment, learning style, learning approach, quality of written questions, quality and setting of OSCE stations and time allowed for each station. The learning outcomes related to cognitive domain were assessed in written assessment; where our focus was to assess the student’s problem solving and critical thinking skills, while outcomes related to clinical skills, procedural skills, communication skills and interviewing skills were assessed in OSCE. The scores taken in both domains were analyzed to compare the domain based performance.

Our written assessment comprised of two papers One 60 item MCQ (single best question) paper, and another eight item SAQ (short answered question) paper. The questions were selected in accordance with table of specifications to ensure content validity. Each MCQ began with a stem and a lead-in statement followed by five options. Maximum marks were 60, with one mark for a correct answer, and no negative marking for a wrong answer. The SAQ paper had eight questions, with five marks for each correct answer and total score was of 40 marks. Test time was one hour and thirty minutes for each paper. Regarding the paper checking MCQ papers were checked manually by pre determined key. SAQ answers sheets were also checked by pre determined structured key, marked by a pair of examiners and average score of the pair was assigned as the final score. Absolute method of standard setting at 50% of cumulative score was set as cut-off value to pass.

Our second assessment tool was OSCE comprised of 10 stations. Each station was allotted 10 marks with the total score of 100. Out of ten, six were interactive to cover the content of counseling, clinical task performance and procedural skills. Faculty members were involved in the construction and assessment of OSCE. Students were evaluated on the basis of their knowledge & skills required to perform the assigned task. The checklist included the main components of the skill being assessed. Global rating scale was applied where required. At the end of assessment students were instructed to fill the questionnaire. The questions and the potential responses were carefully framed through departmental consensus meeting. The questions were selected to assess the student’s perception with respect to both assessment tools like quality of written questions, quality and setting of OSCE stations and time allowed for both assessment methods. Responses were measured by using Likert rating scale.

Data analysis was done by SPSS version 22. Mean and ±SD were drawn. As our female students were 104 and male students were 56, so paired sample test was applied to keep the sample in both groups equal; the total scores of 56 male students were compared with 56 female students selected by stratified sampling technique. To select the 56 female students, all female students (104) were divided in four equal groups (26 in each) and randomly 14 were selected from each group (14X4=56). Correlation between written and OSCE score was estimated by using Logistic regression analysis and student T-test were applied to measure the association of dependent to independent variables. Construct validity of SAQ is estimated by inter-rater reliability and Pearson’s coefficient test. P-value of <0.05 was considered statistically significant.

### RESULTS

In this study 160 students appearing in the end semester pediatric examination were enrolled. Mean and SD of scores obtained in the written examination (MCQ and SAQ) were compared with scores taken on OSCE performance. In our batch male students were 56, while 104 were female students (Female: Male ratio was 1.9:1). Performance of the female students was better in both assessment tools. There was statistically significant difference when the means of their theory score compared with the mean of OSCE score in both gender by independent T-test as shown in Table-I. But when similar comparison done by applying paired sample T-test, statistically significant difference seen only in OSCE performance. (Table-II).

**Table-I:** Comparison Theory and OSCE Exam result (Independent T-Test).

Gender		Sample	Mean	Std. Deviation	P-Value
Theory	Male	56	59.2222	6.34780	0.011
	Female	104	61.7981	8.34976	
OSCE	Male	56	70.1481	9.84061	0.005
	Female	104	73.5962	8.33646	

**Table-II:** Comparison Theory and OSCE Exam result (Paired sample Test).

Gender		Sample	Mean	Std. Deviation	P-Value
Theory	Male	56	59.2222	6.34780	0.122
	Female	56	62.2222	11.29514	
OSCE	Male	56	70.1481	9.84061	0.000005
	Female	56	65.1481	9.84061	

Regarding the comparison of OSCE and written within the same group, that’s female OSCE score compared with their written score and male OSCE score compared with their written score by applying paired sample T-test, statistically significant difference was found in the male group only (Table-III).

**Table-III:** Comparison of means of written & OSCE by gender.

Paired Samples Statistics								

		Mean	Sample	Std. Deviation	95% CI		Correlation	P-value

					Lower	Upper		
Female Result	Theory	61.7981	104	8.34976	-8.15326	-5.24297	0.554	7.4207
	OSCE	73.5692		8.33646				
Male Result	Theory	59.222	56	6.34780	-10.989	-7.196	0.439	0.001
	OSCE	70.1481		9.84061				

Out of 160 students, 150 had participated in the survey and filled the questionnaire. Their responses were shown in Table-IV.

**Table-IV:** Questionnaire to find reasons for discrepancies in scores of written & OSCE.

S. No	Questions	Mode of Assessment	Strongly disagree	Disagree	Don’t know	Agree	Strongly agree
1	Content coverage was adequate	Written	00	10%(15)	00	60%(90)	30%(45)
		OSCE	00	20%(30)	00	60%(90)	20%(30)
2	Students were informed about the format of assessment	Written	00	00	00	30% (45)	70%(105)
		OSCE	00	00	00	30% (45)	70%(105)
3	The questions/ tasks were relevant	Written	10% (15)	10%(15)	10%(15)	50%(75)	20% (30)
		OSCE	00	10%(15)	6%(9)	60%(90)	24% (36)
4	Adequate time was allocated	Written	00	00	00	40% (60)	60% (90)
		OSCE	00	10%(15)	00	80%(120)	10%(15)
5	Construction of questions/ task were clear	Written	4%(6)	4%(6)	8%(12)	64%(96)	20%(30)
		OSCE	00	6%(09)	4%(6)	80%(120)	10%(15)
6	Assessment was focused on testing knowledge based	Written	00	10%(15)	00	60%(90)	30%(45)
		OSCE	00	20%(30)	20%(30)	50%(75)	10%(15)
7	Assessment was focused on testing clinical skills	Written	20%(30)	30%(45)	20%(30)	30%(45)	00
		OSCE	00	6%(09)	4%(6)	70%(105)	20%(30)
8	Motivated further learning	Written	00	00	20%(30)	50%(75)	30%(45)
		OSCE	00	10%(15)	8%(12)	70%(105)	12%(18)
9.	The question/tasks given reflected those that were taught	Written	10%(15)	20%(30)	10%(15)	60%(90)	00
		OSCE	4%(6)	4%(6)	8%(12)	64%(96)	20%(30)
10	Reading past pool is enough to get through examination. (MCQs & OSCE books).	Written	00	20%(30)	00	60%(90)	20%(30)
		OSCE	30%(45)	20%(30)	00	50%(75)	00

## DISCUSSION

End-semester test is good assessment strategy for undergraduate medical students. In our department it works as formative as well as summative, as feedback was given about their deficiencies and marks obtained has been included in the cumulative score as ratio of 10%; hence the students had taken it seriously and considered it as a good preparatory practice. Total 160 students were enrolled. Their scores obtained in the written examination (MCQ and SAQ) were compared with scores taken on OSCE performance. A statistically significant correlation was seen between scores of SAQ and MCQ; students who performed well in MCQ also produced better score in SEQ (r= 0.5, p < 0.01). This correlation indicates that both assessment methods were different but had assessed same cognitive domain. Same observation was drawn from other studies as well.^9^ The correlation between the written tests and OSCE was surprisingly low as both assessment methods are markedly different in terms of construct and content validity. Objective structured clinical examination (OSCE) measures performance-based outcomes, not otherwise measured by traditional assessment tools like written, to validate achievement of required level of competencies expected from our medical graduates.^2^ Though there is some evidence of examiner bias, OSCE has proven to be a reliable and valid mode of assessment for clinical skills. In our study students had secured more score on the OSCE as compared to written assessment. Statistically significant difference was observed when the means of their theory and OSCE scores were compared by independent T-test. This might be because only 10 stations were selected for the OSCE due to shortage of examiners and resources while written assessment comprised 60 item MCQs and eight item SAQ papers with broader content validity. Examiner bias may be another reason to achieve more scores at some stations. In contrast to our findings some studies had concluded that the students secured less scores on the OSCE. In one study 33 target skills were selected for OSCE assessment comprised of 20 stations but only 50% students had demonstrated satisfactory performance on 16 stations only.^10^ This might be because of broader content coverage of OSCE in their study. We observed higher means of scores of female students in written as well in OSCE than male students with statistically significant difference. Such similar observation was also seen in other studies,^11^ but Faisal R, et al. study had shown no difference in the academic performance on gender basis.^12^ According to study of Conger and Long at public institution Florida, males obtained 0.43 less credits than females in the first semester and got even lesser in subsequent semesters. By the end of the sixth semester, males had a cumulative disadvantage of 6.6 credits.^13^ Contrary to the above-mentioned studies, according to studies conducted in Nigeria^14^ and Bangladesh^15^ male students were with higher academic performance than their female counterparts.

As regards the comparison of OSCE and written within the same group by applying paired sample T-test, statistically significant difference was found in the male group only. This observation was further supported by finding statistically significant difference when means of their total scores (written +OSCE) were compared. The difference might be because female students usually chose group discussion and deep learning styles for their studies and achieved better scores in both domains of assessment even with difference in the, content, environment, task, and examiners.^16^ On the other hand, male students are surface or strategic learner achieved higher score in the OSCE only, which has limited content area. Another explanation for better performance of our female students is difference in their nature, as females perceive more stress of their assigned task and that stress enhances their functioning capability. This finding is further supported by study conducted at Sheikh Zayed Medical College. They compared the academic performance in relation to level of stress among undergraduate medical students, and concluded that little stress is necessary to accomplish the task and can improve performance.^17^ Another study showed similar results that Female students show greater anxiety and increased emotional responses to complete the assigned tasks as compared to male students.^18^

Regarding the responses that were collected by distributing the questionnaire, only 50% students agreed that the tasks given were easy to understand and problem based scenarios were considered difficult by 50% respondents. Similar finding was seen in the study of Mehmood H.^19^ Around 20% students showed disagreement that wide area of knowledge was covered by MCQs paper, but 70% agreed that reading past papers and solved MCQs books are enough to get through MCQ papers. This indicates that they were using the surface learning approach and might be the reason of securing less scores in the written assessment. Regarding OSCE examination 70% students replied positively that it was good tool to assess clinical skills. Around 80% also agreed that OSCE had played motivational role for their preparation. These findings are also supported by other studies as well.^20^ All the students agreed that they were informed about the assessment tools and time allocation was adequate in both assessment tools.

The observations of this study proves the hypothesis that there is difference in domain based achievement among the medical graduate students. Those students who are deep learner with clear concept achieved higher score in both domain as compared to surface learner.

### Limitation of the study

Findings of our study cannot be generalized as study was done at pediatric department LUMHS and not at other departments or institutions.. Scores of written and OSCE were compared, but affective domain which is also one of the important component was not included. Item analysis or Cronbach’s alpha if item deleted was not performed for the MCQ’s assessment.

## CONCLUSION

There was difference in the domain based achievement. Students performed better in OSCE than in written assessment. In OSCE they have shown unsatisfactory performance for some important key competencies like clinical examination, task performance methods and procedural skills. The performance of Female students was comparatively better.

## RECOMMENDATION

- Teaching institutions should modify the teaching methodology and assessments tools, and make the assessment tools a strong barrier so that no one can pass through until or unless desired competencies achieved.
- There should be periodic faculty training on item writing, and construction of OSCE for improving the reliability and validity, as essential learning outcomes can only be assessed with valid and reliable assessment tools.
- QEC should arrange the Periodic feedback sessions with the faculty on their performances.
- DME should periodically revise the curriculum including table of specification to check the credibility of assessment tools in relation to learning outcomes.

### Authors’ Contributions

**SM:** Principal investigator, Data collection and analysis, Writing Manuscript analysis and interpretation of data and responsible for accuracy and integrity of the research work.

**SH:** Research supervisor, Contribution for conception, design and data interpretation, Final reversion of draft.
